# Accumulation of meningeal lymphocytes correlates with white matter lesion activity in progressive multiple sclerosis

**DOI:** 10.1172/jci.insight.151683

**Published:** 2022-03-08

**Authors:** Shanzeh M. Ahmed, Nina L. Fransen, Hanane Touil, Iliana Michailidou, Inge Huitinga, Jennifer L. Gommerman, Amit Bar-Or, Valeria Ramaglia

**Affiliations:** 1 Department of Immunology, University of Toronto, Toronto, Ontario, Canada.; 2 Department of Neuroimmunology, Netherlands Institute for Neuroscience, Meibergdreef, Amsterdam, Netherlands.; 3 Department of Pathology, University Medical Center Utrecht, Utrecht, Netherlands.; 4 Department of Neurology and Center for Neuroinflammation and Neurotherapeutics, Perelman School of Medicine, University of Pennsylvania, Philadelphia, Pennsylvania, USA.; 5 Department of Clinical Genetics, Leiden University Medical Center, Einthovenweg, Leiden, Netherlands.; 6 Swammerdam Institute for Life Sciences, University of Amsterdam, Amsterdam, Netherlands.

**Keywords:** Immunology, Adaptive immunity, Demyelinating disorders, Multiple sclerosis

## Abstract

Subpial cortical demyelination is an important component of multiple sclerosis (MS) pathology contributing to disease progression, yet mechanism(s) underlying its development remain unclear. Compartmentalized inflammation involving the meninges may drive this type of injury. Given recent findings identifying substantial white matter (WM) lesion activity in patients with progressive MS, elucidating whether and how WM lesional activity relates to meningeal inflammation and subpial cortical injury is of interest. Using postmortem FFPE tissue blocks (range, 5–72 blocks; median, 30 blocks) for each of 27 patients with progressive MS, we assessed the relationship between meningeal inflammation, the extent of subpial cortical demyelination, and the state of subcortical WM lesional activity. Meningeal accumulations of T cells and B cells, but not myeloid cells, were spatially adjacent to subpial cortical lesions, and greater immune cell accumulation was associated with larger subpial lesion areas. Patients with a higher extent of meningeal inflammation harbored a greater proportion of active and mixed active/inactive WM lesions and an overall lower proportion of inactive and remyelinated WM lesions. Our findings support the involvement of meningeal lymphocytes in subpial cortical injury and point to a potential link between inflammatory subpial cortical demyelination and pathological mechanisms occurring in the subcortical WM.

## Graphical abstract

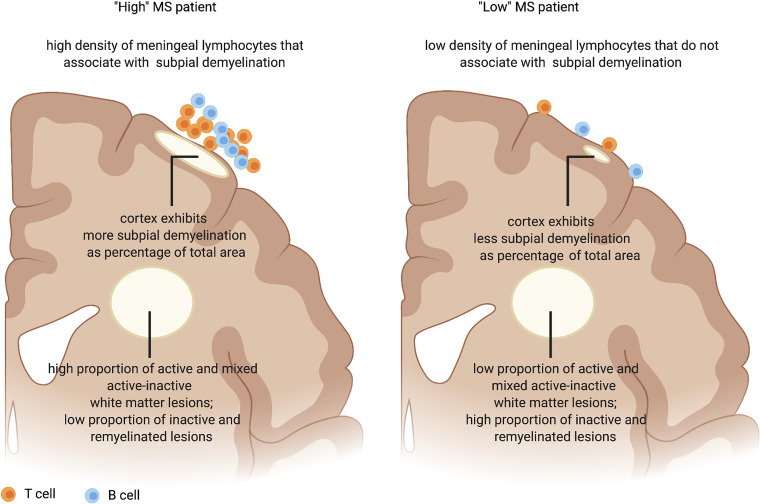

## Introduction

Subpial (type III) cortical lesions have emerged as an important component of multiple sclerosis (MS) pathology (1–7). Extensive subpial cortical demyelination has been reported in earlier stages of MS (4, 5, 8) and appears to reach maximal burden in the latter progressive stage of the disease (1–3, 6, 7). Subpial changes are not limited to demyelination but also include neuronal (9, 10) and axonal (11) damage. Importantly, the extent of cortical injury is now considered to be a major contributor to disease progression in MS, including physical (12) and cognitive (13, 14) impairments. Understanding the mechanism(s) driving subpial cortical pathology is critical to guiding development of therapies for progressive MS.

The absence of major blood-brain barrier (BBB) disturbances (15), a paucity of parenchymal immune cell infiltration (1, 2), and the lack of significant terminal complement activation (16) within subpial cortical demyelinating lesions suggest that this form of cortical injury either proceeds independently of inflammation or involves inflammatory mechanisms that differ substantially from those underlying classic deep white matter (WM) demyelinating lesions (reviewed in refs. 17, 18). One theory supporting a key role of immune cells in subpial cortical pathology proposes that this injury may be driven by a compartmentalized immune response that takes place within the inflamed meninges of brains that have a relatively intact BBB (4, 5, 9, 11, 19–29). How subpial cortical injury associated with meningeal inflammation may relate to WML activity remains unknown, although the presence of meningeal inflammation has not been found to relate to either the number of WM lesions (WMLs; ref. 9) or the extent of WM demyelination (measured as the percentage of demyelination of total WM in whole-brain coronal slices) (20). Such a relation is of interest, particularly in view of recent pathological findings showing substantial lesion activity in patients with progressive MS (30). Comprehensive analysis of the autopsy cohort of the Netherlands Brain Bank (NBB), which included 3188 tissue blocks containing 7562 MS lesions from 182 brain donors with longstanding disease (>29 years), showed that 57% of all lesions were either active or mixed active/inactive and that 78% of all patients had mixed active/inactive lesions present. Notably, patients with a higher proportion of mixed active/inactive lesions at the time of death had a more severe disease course. Here, we investigated the correlation between the extent of meningeal T cell, B cell, and myeloid cell accumulation and associated subpial demyelination with subcortical WML activity, measured as the proportion of active, mixed active/inactive, inactive, and remyelinated WMLs derived from all available archived FFPE tissue blocks (range, 5–72 blocks; median, 30 blocks) for each of 27 patients with progressive MS collected at rapid autopsy from the NBB. We confirmed that meningeal immune cells were enriched in patients with MS compared with controls. Stratifying patients with MS into subgroups with high versus low numbers of meningeal immune cells revealed that patients with a high number of meningeal T cells and B cells exhibited not only a higher proportion of subpial gray matter that did not stain positive for myelin, but also a greater proportion of active and mixed active/inactive lesions in the subcortical WM and a lower proportion of inactive and remyelinated WMLs. Of interest, meningeal myeloid cell numbers did not correlate with either gray matter or WM pathology. While the relationship between gray matter subpial lesions and meningeal inflammation is already appreciated (9, 11, 20, 25), our data connect some aspects of WM pathology with lymphocyte residence in the subarachnoid space in progressive MS.

## Results

### A high density of meningeal T and B lymphocytes is linked to cortical subpial demyelination in progressive MS cases.

**Figure 1:**
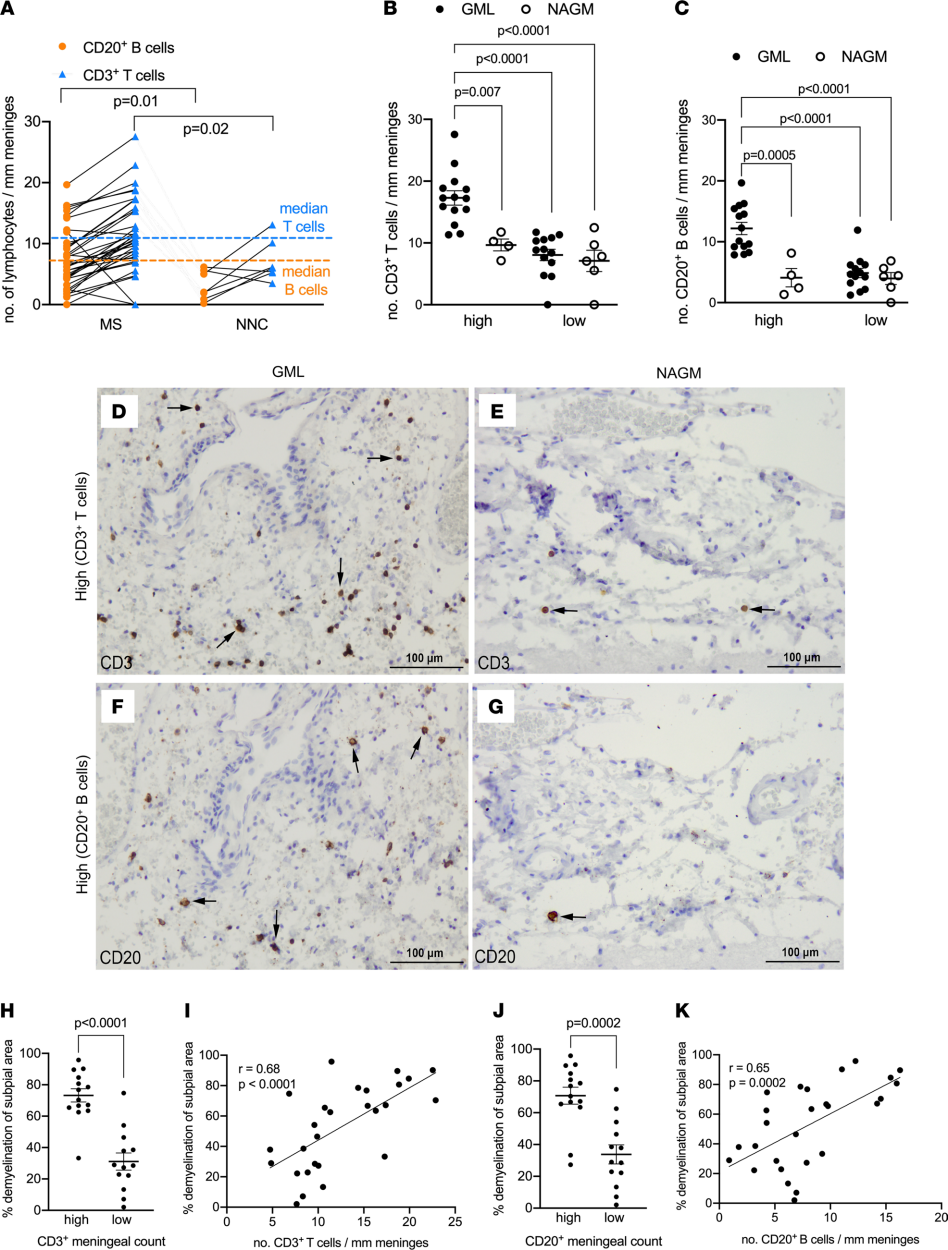
Meningeal T cells and B cells are enriched in MS and topographically linked to cortical subpial demyelination. (**A**) Quantification of meningeal CD20^+^ B cell count and CD3^+^ T cell count in 27 MS donors and 9 nonneurological controls (NNCs) showing enrichment in MS donors versus NNCs. The median for meningeal CD20^+^ B cell count (orange dotted line) and meningeal CD3^+^ T cell count (blue dotted line) was used to stratify MS donors in high versus low meningeal B or T cell count. (**B**) Quantification of meningeal CD3^+^ T cell count showing significant enrichment of T cells in meninges adjacent to GML versus NAGM in MS donors with high (*n* = 14) but not low (*n* = 13) meningeal T cell count. (**C**) Quantification of meningeal CD20^+^ B cell count showing significant enrichment of B cells in meninges adjacent to GML versus NAGM in MS donors with high (*n* = 14) but not low (*n* = 13) meningeal T cell count. (**D**–**G**) Representative immunohistochemical staining for CD3 (**D** and **E**, arrows) and CD20 (**F** and **G**, arrows) in meninges adjacent to subpial GML or NAGM in MS donors with high CD3^+^ or CD20^+^ meningeal cell count. (**H**) Quantification of the proportion of subpial gray matter that did not stain positive for myelin in MS donors with high versus low CD3^+^ T cell meningeal count. (**I**) Spearman’s correlation coefficient between meningeal CD3^+^ T cell count and the proportion of subpial gray matter that did not stain positive for myelin. (**J**) Quantification of the proportion of subpial gray matter that did not stain positive for myelin in MS donors with high versus low CD20^+^ B cell meningeal count. (**K**) Spearman’s correlation coefficient between meningeal CD20^+^ B cell count and the proportion of subpial gray matter that did not stain positive for myelin. In **A**–**C** each data point represents mean cell count (mean ± SD) in all fields analyzed per case. Statistically significant differences determined by nonparametric Mann-Whitney test (**A**, **H**, and **J**) or nonparametric Kruskal-Wallis test followed by Dunn’s correction for multiple comparisons (**B** and **C**). Scale bars: 100 μm (**D**–**G**).

To assess the extent of meningeal inflammation and its relationship with cortical subpial demyelination, the density of meningeal lymphocytes and their topographic association with subpial demyelination were analyzed in FFPE tissue blocks dissected from the supratentorial cortex of 27 MS cases compared with 9 controls. As expected, the numbers of CD3^+^ T cells and CD20^+^ B cells per unit length of the meninges were significantly increased in MS cases compared with control cases (Figure 1A). In addition, a significant positive correlation between the number of T cells and B cells measured in each donor per unit length of meninges was observed (Supplemental Figure 1; supplemental material available online with this article; https://doi.org/10.1172/jci.insight.151683DS1). Considering the large dynamic range of the meningeal count for CD3^+^ T cells (range: 4.74–22.87 cells/mm meninges) and CD20^+^ B cells (range: 0.89–16.27 cells/mm meninges) in MS cases, the median was chosen as a cutoff to stratify the MS cases in those with high (above the median) or low (below the median) meningeal lymphocyte count (Figure 1A — 10.72 cells/mm for T cells; 7.30 cells/mm for B cells). Similar to other studies that have demonstrated a correlation between the presence of immune cell aggregates in the MS meninges and demyelination in the adjacent subpial brain compartment (9, 11, 20, 25), when comparing areas of subpial gray matter lesions (GMLs) to normal-appearing gray matter (NAGM) within patients (Supplemental Figure 2), we found that T and B cell infiltrates were selectively enriched in areas of the meninges that were adjacent to subpial lesion areas compared with NAGM in the MS group with a high count of meningeal lymphocytes (Figure 1, B–G, and Supplemental Figure 3). It should be noted that although increased in density, the immune cell infiltrates were never found as aggregates resembling the follicle-like structures previously described (11).

Using the “high versus low” stratification for the extent of meningeal inflammation, we next determined whether the density of T cells and B cells in the meninges was associated with clinical metadata parameters or cortical gray matter demyelination at autopsy. With respect to the former, we found that age at the time of death and disease duration were not significantly different for patients with MS with high versus low meningeal T or B cell counts (Supplemental Figure 4, A and B, and Supplemental Table 1). With respect to the latter, using the entire archived collection of tissue blocks per donor, which included a comparable number of dissected blocks from donors with high versus low meningeal cell counts, we found that donors with high meningeal lymphocyte counts also had a significantly higher number of overall cortical GMLs compared with donors with low meningeal lymphocyte counts (Supplemental Figure 5, A and B). However, in terms of the types of GMLs, the proportion of leukocortical (Supplemental Figure 5, C and D), intracortical (Supplemental Figure 5, E and F), or subpial GMLs (Supplemental Figure 5, G and H) did not differ between MS donors with high versus low meningeal inflammation. Given that subpial cortical GMLs can extend over large areas also affecting multiple gyri, we next measured the proportion of subpial gray matter that did not stain positive for myelin in the same tissue blocks in which we assessed the meningeal lymphocyte counts. We found that donors with high meningeal lymphocyte counts also had a significantly higher proportion of subpial gray matter that did not stain positive for myelin compared with donors with low meningeal lymphocyte counts. In addition, we assessed the relationship between meningeal lymphocytes and the extent of subpial demyelination on a per-donor basis by a nonparametric Spearman’s correlation analysis and observed that the density of meningeal T cells and B cells positively correlated with the proportion of subpial gray matter that did not stain positive for myelin in the MS cases (Figure 1, H–K). In summary, while the density of meningeal T and B lymphocytes was not associated with donor clinical metadata (age of death, mean disease duration), donors with a high density of meningeal T and B lymphocytes exhibited a greater extent of subpial demyelination compared with donors with low meningeal T and B lymphocytes.

### Meningeal lymphocytes are associated with WML activity in progressive MS.

**Figure 2:**
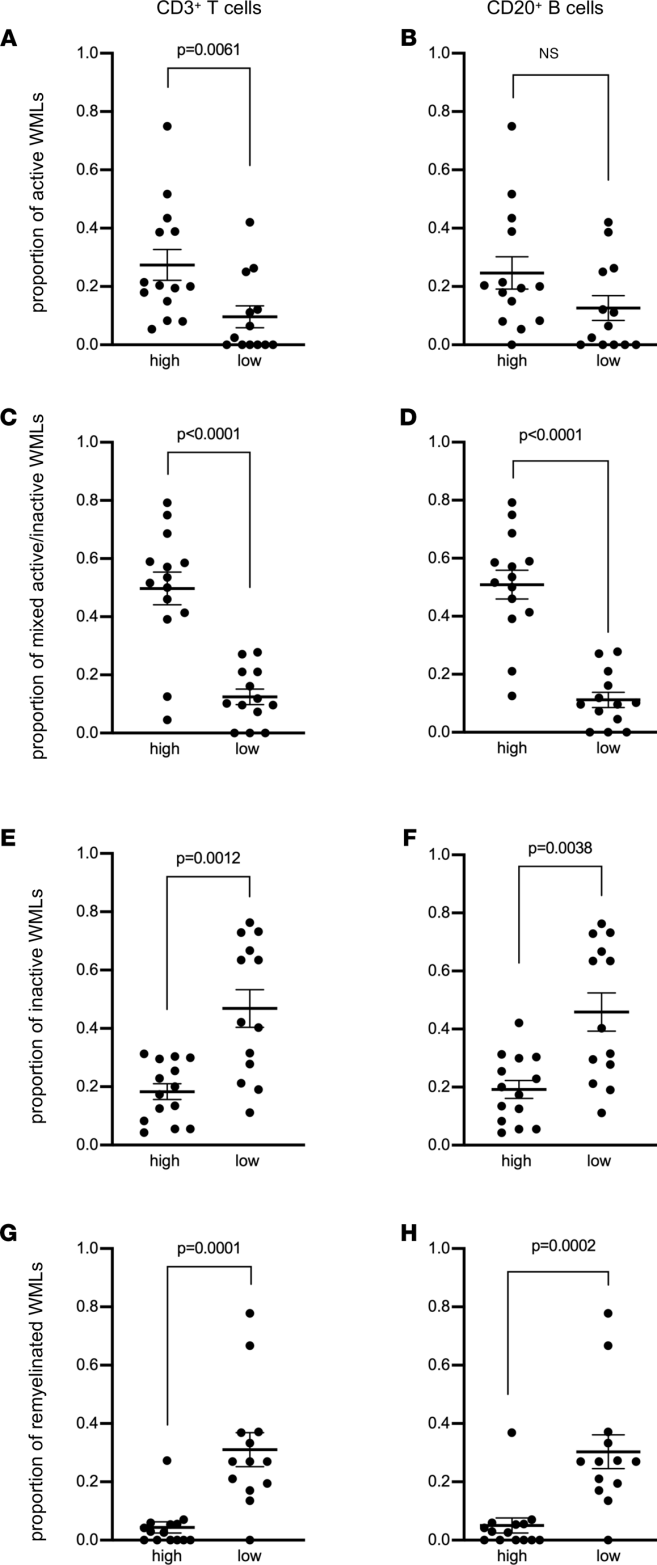
Enrichment of meningeal T cells and B cells is linked to subcortical white matter lesion activity. Quantification of (**A** and **B**) the proportion of active white matter lesions (WMLs), (**C** and **D**) mixed active/inactive WMLs, (**E** and **F**) inactive WMLs, and (**G** and **H**) remyelinated WMLs in MS donors with high (*n* = 14) versus low (*n* = 13) meningeal CD3^+^ T cells (**A**, **C**, **E**, and **G**) or CD20^+^ B cell (**B**, **D**, **F**, and **H**) count. Each data point represents the proportion of WMLs in all tissue blocks analyzed per case (range 5–72 blocks, median: 30 blocks per donor). Statistically significant differences were determined by the nonparametric Mann-Whitney test.

Although an association between meningeal inflammation and subpial gray matter injury has been described in multiple studies (4, 5, 9, 11, 19–29), 2 studies have looked at whether a relationship exists between meningeal inflammation and the number of WMLs (11) or the percentage of demyelination in the subcortical WM (20), and they found no link. However, the relationship between meningeal inflammation and the type of lesion activity in the subcortical WM has not been examined to our knowledge. We first asked whether the density of meningeal lymphocytes was associated with the proportion of active, mixed active/inactive, inactive, or remyelinated WMLs obtained from the entire archived collection of tissue blocks per donor. Although MS donors with high versus low meningeal T or B cell count did not differ with respect to the total number of subcortical WMLs (Supplemental Figure 6, A and B) as seen in another study (11), we found that donors with a high density of meningeal T or B cells had a significantly greater proportion of active (Figure 2, A and B) and mixed active/inactive WMLs (Figure 2, C and D). In contrast, donors with a high meningeal T or B cell count exhibited a significantly lower proportion of inactive (Figure 2, E and F) and remyelinated WMLs (Figure 2, G and H) compared with donors with low lymphocyte meningeal count. Of note, active and mixed active/inactive lesions (unlike inactive lesions) harbored perivascular cuffing of T and B cells (Supplemental Figures 7 and 8), which have been linked to enriched meningeal immune cells in another cohort of patients with MS (20). Altogether, these data revealed a link between the enrichment of meningeal T and B cells and the WML activity in the subcortical compartment.

### Absence of a numerical or topographical association between meningeal myeloid cell density and subpial or WM pathology in progressive MS.

**Figure 3:**
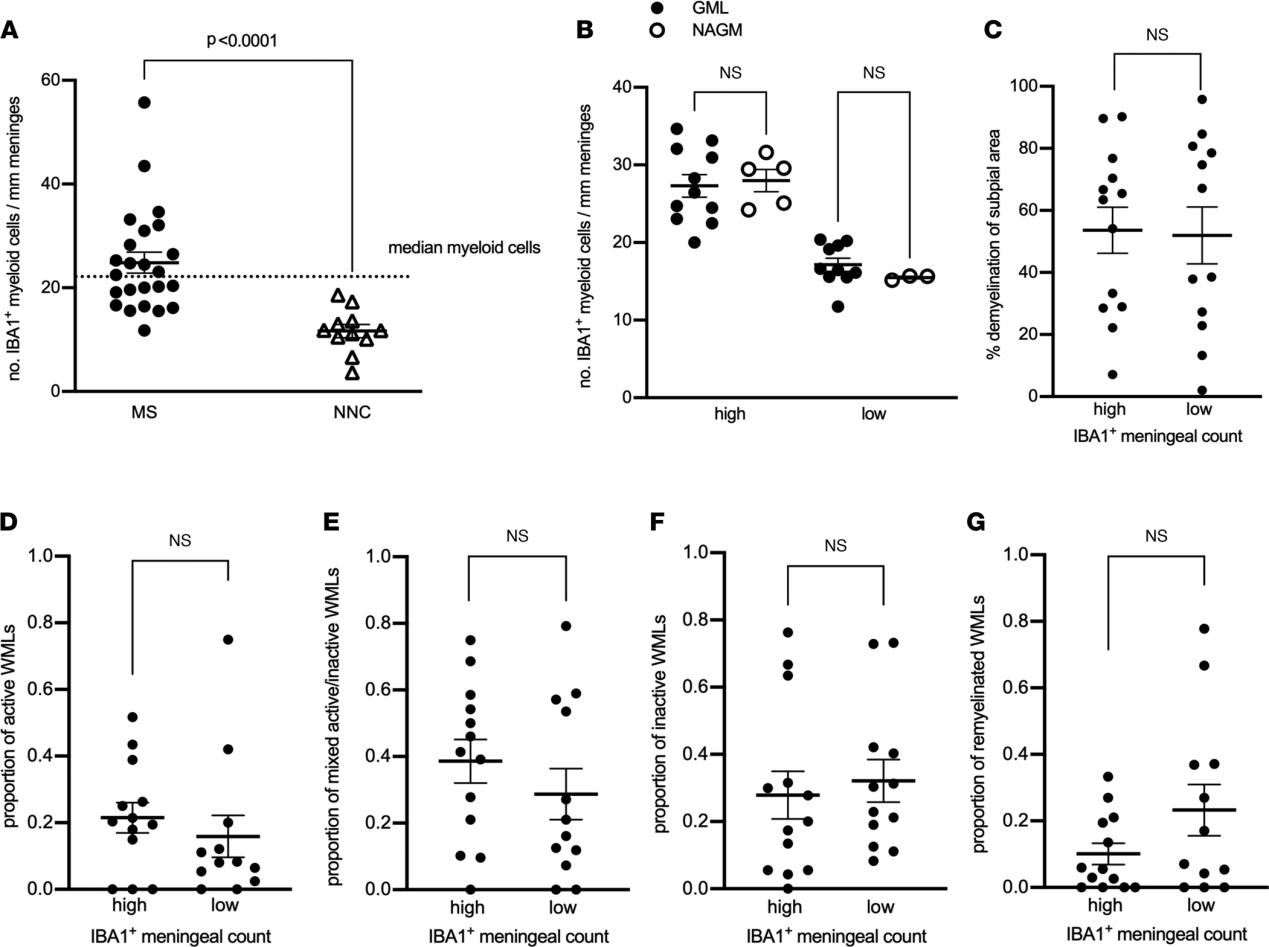
Meningeal myeloid cells are enriched in MS but not topographically linked to subpial demyelination or to the extent of cortical subpial demyelination or to subcortical white matter lesion activity. (**A**) Quantification of meningeal IBA1^+^ myeloid cell count in MS donors and nonneurological controls (NNCs) showing enrichment in 27 MS donors compared with 9 NNCs. The median for meningeal IBA1^+^ myeloid cell count (shown by the dotted line) was used to stratify MS donors in high versus low meningeal myeloid cell count. (**B**) Quantification of meningeal IBA1^+^ myeloid cell count showing no significant changes in the number of meningeal myeloid cells adjacent to GML versus NAGM in MS donors with high (*n* = 14) or low (*n* = 13) meningeal myeloid cell count. (**C**) Quantification of the proportion of subpial gray matter that did not stain positive for myelin in MS donors with high versus low meningeal IBA1^+^ myeloid cell count. (**D**–**G**) Quantification of (**D**) proportion of active white matter lesions (WMLs), (**E**) mixed active/inactive WMLs, (**F**) number of inactive WMLs, and (**G**) number of remyelinated WMLs in MS donors with high versus low meningeal IBA1^+^ myeloid cell count. Each data point represents the proportion of WMLs in all tissue blocks analyzed per case (range 5–72 blocks, median: 30 blocks per donor). In **A** and **C**–**G**, statistically significant differences were determined by nonparametric Mann-Whitney test. In **B**, statistically significant differences were determined by nonparametric Kruskal-Wallis test followed by Dunn’s correction for multiple comparisons.

We next assessed whether similar associations between meningeal lymphocyte density and brain pathology were observed for meningeal myeloid cells by staining the same tissues with an antibody that detects ionized calcium binding adaptor molecule 1 (IBA1) on microglia/macrophages and an antibody that detects CD68 on the lysosomal membrane of cells (31). Although CD68 is expressed by a number of cell types, it is enriched on macrophages and other mononuclear phagocytes and therefore used to identify myeloid cells, particularly those with ongoing phagocytic activity (32). Since a nonparametric Spearman’s correlation analysis on a per-donor basis showed that the number of CD68^+^ cells per millimeter length of meninges had a strong positive correlation with the number of IBA1^+^ cells per millimeter length of meninges (Supplemental Figure 9), we subsequently report only on the analysis of the IBA1^+^ myeloid cells. The density of IBA1^+^ meningeal cells was significantly increased in MS cases compared with control cases (Figure 3A). Similar to the findings for T and B cells, we also found a wide dynamic range of the meningeal count of IBA1^+^ myeloid cells (range: 11.76–34.64 cells/mm meninges) (CD68^+^ cell range: 17.01–62.57 cells/mm meninges; median 27.89 cells/mm meninges) in MS cases. Therefore, the IBA1^+^ median (22.47 cells/mm meninges) was chosen as the cutoff to stratify MS cases with high versus low myeloid meningeal density (Figure 3A). As we observed for lymphocytes, neither the age at the time of death nor disease duration differed in myeloid high versus low subgroups (Supplemental Figure 10 and Supplemental Table 1).

Unlike what we observed for meningeal lymphocytes, quantification of the number of myeloid cells in meningeal areas adjacent to subpial GMLs or to NAGM showed no spatial relation to GMLs in either group (high versus low myeloid cell density) (Figure 3B and Supplemental Figure 11). In addition, the proportion of subpial gray matter that did not stain positive for myelin did not differ between high and low myeloid cell density donor subgroups (Figure 3C). Similarly, MS donors with high versus low meningeal myeloid cell densities exhibited a similar total number of subcortical WMLs (Supplemental Figure 12), as well as comparable proportions of active (Figure 3D), mixed active/inactive (Figure 3E), inactive (Figure 3F), or remyelinated WMLs (Figure 3G). Altogether, these data showed that although myeloid cells were enriched in the meninges of patients with progressive MS, they were not linked to subpial cortical demyelination nor subcortical WML activity.

### The density of meningeal T and B cells directly correlates with the extent of subpial GMLs as well as active and mixed active/inactive WMLs in progressive MS.

**Figure 4:**
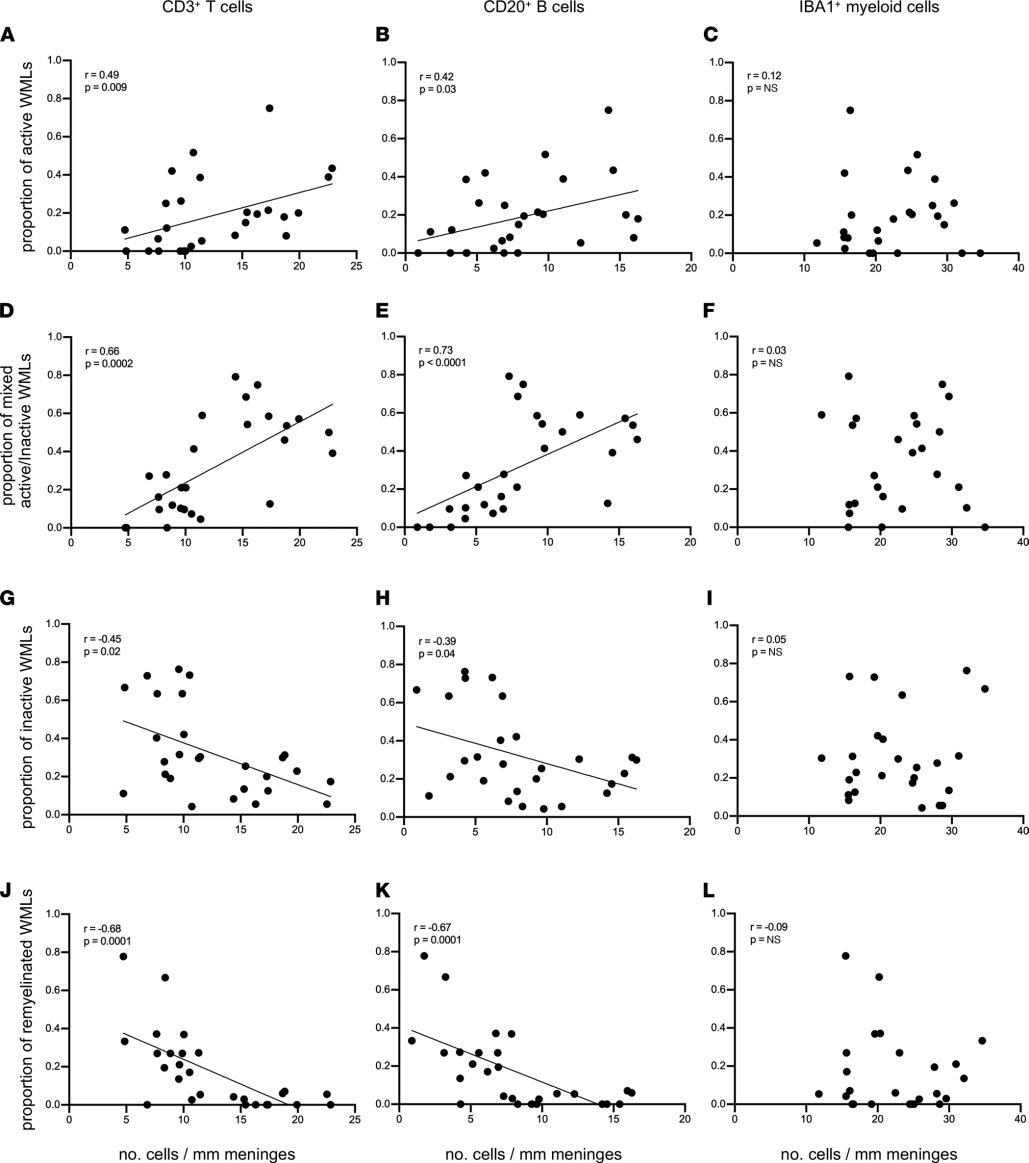
The density of meningeal CD3^+^ T cells and CD20^+^ B cells but not IBA1^+^ myeloid cells correlates with subcortical white matter lesion activity. Spearman’s correlation coefficient between meningeal CD3^+^ T cell, CD20^+^ B cell, and IBA1^+^ myeloid cell count with proportion of active white matter lesions (WMLs) (**A**–**C**), proportion of mixed active/inactive WMLs (**D**–**F**), proportion of inactive WMLs (**G**–**I**), and proportion of remyelinated WMLs (**J**–**L**) in each of 27 MS donors. Each data point represents the proportion of WMLs obtained from all tissue blocks analyzed per case (range 5–72 blocks, median: 30 blocks per donor) and the mean meningeal cell count in all fields analyzed per case.

To assess the relationship between meningeal immune cells and brain pathology on a per-donor basis, we performed a nonparametric Spearman’s correlation analysis for MS cases. We observed that the density of meningeal T and B cells positively correlated with the proportion of active and chronic active/inactive WMLs but correlated negatively with the number of remyelinated lesions. In contrast, we observed no correlation, positive or negative, between the density of meningeal myeloid cells and the proportion of WMLs (Figure 4, A–L). These data suggest a link between the density of meningeal T cells and B cells (but not myeloid cells) with subpial cortical pathology and active WM pathology as well as poor repair processes in WMLs.

## Discussion

In this study, we established a link between meningeal lymphocytes, subpial cortical demyelination, and subcortical WML activity. Specifically, progressive MS brain donors with the highest densities of B and T cells in the meninges not only exhibited greater subpial cortical demyelination (as shown previously by others; refs. 9, 11, 20, 25) but also had greater proportions of active and mixed active/inactive lesions in the subcortical WM. These data point to a connection between pathological mechanisms occurring in the subcortical WM and inflammatory subpial cortical demyelination. In addition, an important implication of our findings is that pathological studies aimed at assessing potential relationships between features of meningeal inflammation and subpial cortical tissue should focus on pathological material in which the presence of subcortical WML activity is documented.

In contrast to meningeal T cells and B cells, we noted that although myeloid cell densities in the meninges were increased in MS brains compared with control brains, myeloid cell abundance in meninges of patients with MS was unchanged when comparing regions proximate to GMLs or NAGM. This suggests that meningeal myeloid cells are either not involved in the subpial cortical injury of MS or that they only exert pathological activity in the presence of neighboring T cells and/or B cells. Another possibility that our findings do not preclude is that although the numbers of meningeal myeloid cells did not differ between regions proximate to GMLs or NAGM, they may have differed in their functional response profile. Last, since our study is necessarily cross-sectional, it is possible that myeloid cells perform important function(s) in the meninges that precede(s) the entry of lymphocytes. Longitudinal assessment of the presence of different types of meningeal immune cells at different stages of the disease would require advanced PET imaging and MRI techniques (33) and/or the application of a relevant animal model that captures meningeal inflammation and other aspects of MS pathology, as we have described before (34, 35).

Although we did not detect particularly dense immune cell aggregates (or “follicle-like” structures) in the meninges of any of the patients in our progressive MS cohort, our quantitative analysis of meningeal immune cells demonstrated numbers of T cells, B cells, and myeloid cells that were very much in keeping with quantification of these cells reported in prior pathological studies, which included detection of B cell–rich immune cell aggregates or diffuse meningeal inflammation in patients with progressive forms of the disease (20, 22). The absence of follicle-like structures in these tissues may reflect a technical issue — importantly, the NBB dissection protocol involves stripping of the meninges from the brain at autopsy, such that only meninges that are trapped within the sulci remain in the brain tissue blocks. Although the tissue blocks in our hands did have a sufficient amount of intact meninges (about 70% of surface area analyzed contained meninges) to allow for the quantification of meningeal immune cells, this preparation could explain why follicle-like meningeal structures were not detected in the tissue we examined. Another possibility for the lack of B cell–rich immune cell aggregates in our cohort could be the older age at death compared with other studies that have identified such meningeal immune cell aggregates. In our study, the age at the time of death of patients with MS ranged from 41 to 81 years (median: 58 years). Magliozzi et al. (11) found that meningeal immune cell aggregates could be detected only in patients with younger age at death (median: 42 years in follicle-positive versus median: 55 years in follicle-negative).

Our findings of a selective association between increased numbers of meningeal T cells and B cells with the extent of subpial GMLs do not prove causality or direction of causality, though they are nonetheless consistent with a potential contribution of T cell– and/or B cell–secreted factors in propagating subpial cortical injury. While we did not find a link between the extent of meningeal inflammation and the number of subpial cortical lesions, it would be relevant to investigate whether a link exists between the extent of meningeal inflammation and the activity of the subpial cortical lesions. However, we were unable to address this question in our cohort since it did not include subpial cortical lesions with evidence of activity (i.e., a rim of IBA1^+^ or CD68^+^ cells), likely because the cohort used for the studies described here included donors with longstanding disease. Therefore, this question should be addressed in a cohort of donors with shorter disease duration. Both T cells and B cells can release factors that may be toxic to neural tissue, and for B cells, such effects may be antibody related or antibody independent (36). In this regard, of interest are reports that secreted products of B cells derived from patients with MS (but not from controls) can induce apoptotic cell death in oligodendrocytes (37) as well as neurons (38), a toxicity that was neither antibody nor complement dependent.

With respect to the link between meningeal inflammation and WMLs, it is possible that classical WM focal perivascular inflammation and meningeal inflammation in MS are independent and simply overlap temporally in brains while there is ongoing disease activity. Prior pathological studies considering such a relationship have reported that the degree of WM inflammation (defined by the distribution of MHC class II^+^ microglia and/or macrophages across the lesion) did not appear to differ between secondary progressive MS donors with or without meningeal cell aggregates (11). The same study, however, showed that donors with more dense meningeal immune cell aggregates did tend to have substantially more B cells and plasma cells in their perivascular WML cuffs, while brains with little meningeal inflammation exhibited only occasional B cells and plasma cells in their perivascular WML cuffs (11). Indeed, we also confirmed that active or mixed active/inactive WMLs, unlike inactive WMLs, did show perivascular cuffs of B and T cells. More recent studies have shown a link between inflammation in the 2 compartments. Fransen et al. (39) reported that donors with enriched CD20^+^ B cells in the meninges overlaying the brainstem also had enriched CD20^+^ B cells in the perivascular cuffs within lesions in the brainstem. Reali et al. (40) reported that the density of CD20^+^ B cells in the spinal leptomeninges was correlated with the density of WM perivascular CD3^+^ and CD20^+^ lymphocytes.

If a causal link exists between meningeal inflammation and WML, 2 models may be envisioned. According to one model, destruction of myelin and axons by microglia/macrophages within active or mixed active/inactive WMLs could release myelin and axonal antigens that could potentially find their way to the draining cervical lymph nodes (perhaps via the glymphatic pathway; ref. 41). After priming and polarizing events occurring in the lymph nodes, activated lymphocytes more efficiently traffic into the CNS, including the meninges. It is also noteworthy that investigation of cells of the B cell lineage (B cells, plasmablasts, and plasma cells) isolated from different subcompartments of the inflamed MS CNS (meningeal, parenchymal WM, and cerebrospinal fluid) demonstrated shared B cell clones across these compartments in the same brains and suggested that the majority of the secondary diversification events (affinity maturation and class switch) are occurring within the draining cervical lymph node (42). Once in the CNS, lymphocytes can then take up residence in the meninges, perhaps via entry through the blood-meningeal barrier (43).

More recently, the discovery of the existence of functional lymphatic vessels in the meninges (44, 45) has indicated a relevant link between the CNS and peripheral immune system, perhaps providing a form of communication between these different compartments (meninges, brain parenchyma, and lymph nodes). Indeed, experimental studies have shown that tracers and proteins injected into the brain parenchyma and/or the cerebrospinal fluid find their way to the cervical lymph nodes (45–47). Meningeal lymphatics seem to be key players in this communication since they assist in the drainage of cerebrospinal fluid components and meningeal immune cells, enabling immune cells to enter draining lymph nodes in a CCR7-dependent manner (48). That this communication is at play during neuroinflammation has been shown in the experimental autoimmune encephalomyelitis (EAE) model of MS where decreasing lymphatic drainage, using a Visudyne-based approach, diminished acquisition of encephalitogenic properties by antigen-specific T cells, with resulting improvement of clinical symptoms of EAE (48).

Once established in the meningeal compartments, lymphocytes could secrete byproducts that are potentially inflammatory, cytotoxic, and myelinotoxic. These noxious mediators can occupy the cerebrospinal fluid circulating within the subarachnoid space from which they can cross the pial membrane and diffuse into the underlying gray matter, resulting in subpial GMLs, as supported by studies that have correlated high cortical lesion load with proinflammatory cytokines (IFN-γ, TNF, IL-2, and IL-22) (49, 50), molecules related to sustained B cell activity and lymphoid neogenesis (CXCL13, CXCL10, LT-α, IL-6, and IL-10) (49), B cell survival factors (BAFF), factors indicative of BBB leakage (fibrin, complement and coagulation factors), and iron-related proteins (free-hemoglobin and haptoglobin) in the CSF (28).

An alternative possibility is that damage to myelin and axons in active and mixed active/inactive WMLs could trigger retrograde degeneration propagating backward toward cortical neurons, resulting in demyelination and neurodegeneration giving rise to GMLs. Consistent with the idea that WMLs may drive the formation of GMLs, several cross-sectional MRI studies have reported significant correlations between the total gray matter volume and the total WM volume in T1- and T2-weighted lesions (51–54). Moreover, several longitudinal MRI studies have shown an association between the volume of WMLs and the loss of gray matter volume (55), ventricular enlargement (56), and upstream gray matter atrophy of the visual cortex (57). In this model, the primary formation of GMLs is followed by the secondary recruitment of immune cells to the portion of the meninges adjacent to the GML.

In summary, our findings indicate that there is some form of communication between WMLs and meningeal inflammation. Future studies elucidating the significance of such a relationship between these CNS subcompartments will be important to better understand the mechanisms of cortical injury in progressive MS and how these may be targeted therapeutically. To this end, multiplexed tools to phenotype cells in situ in postmortem brain tissue, as we have done before for the brains of patients who died with MS (58) and as others have done for brains from patients who died with COVID-19 (59), are ideal to phenotype immune cells in the meninges vis a vis subpial cortical lesions and are the current focus of our ongoing studies. In addition, high-throughput, unbiased analysis of isolated meninges will uncover hits that can guide our understanding of the cell-type composition and pathways that are at play in the meninges of patients with progressive MS with a high proportion of mixed active/inactive WMLs (and enriched meningeal immune cells) versus those with a high proportion of inactive WMs (and a paucity of meningeal immune cells).

## Methods

### Postmortem tissue retrieval and inclusion criteria.

Tissue blocks for this study were obtained from the NBB (Amsterdam, Netherlands). For the characterization of meningeal immune cells, sample tissues from 27 donors with progressive (primary progressive or secondary progressive) MS were selected based on the presence of meninges adjacent to cortex in the tissue blocks. For the characterization of MS subcortical WMLs, all available archived FFPE tissue blocks for each of 27 patients with MS (range 5–72 blocks, median: 30 blocks per donor) were analyzed (see Supplemental Table 1). Tissue blocks were dissected based on the identification of lesions as guided by macroscopical examination and/or by postmortem MRI (since 2001) of 1 cm thick coronal brain slices (30). The tissue blocks used for the analysis of meningeal inflammation and subpial demyelination performed in this study were dissected from the supratentorial cortex at locations that included the occipital, parietal, temporal, or frontal lobes. Donors with the myelocortical (60) variant of MS were not included in this study.

Detailed clinical-pathological and demographic data of all donors are provided in Supplemental Table 1. The age at the time of death of patients with MS ranged from 41 to 81 years (median: 58 years) with a mean postmortem delay of 8 hours and 31 minutes (SD, ±1 hour 42 minutes). The age at the time of death of the nonneurological controls ranged from 49 to 99 years (median: 63.5 years) with a mean postmortem delay of 9 hours 18 minutes (SD, ±8 hours 37 minutes). Race and ethnicity of the donors are not available in the clinical records and therefore not reported here. The clinical diagnosis of MS and its clinical course were determined by a certified neurologist and confirmed by a certified neuropathologist based on the neuropathological analysis of the patients’ brain autopsy.

### Neuropathological techniques and immunohistochemistry.

For the classification of cortical GMLs, sections were stained by immunohistochemistry for the proteolipid protein (PLP) marker of myelin. For the identification of WMLs, sections were stained with H&E, Luxol fast blue (LFB) marker of myelin lipids, and Bielschowsky silver stain of axons. WML demyelinating and innate inflammatory activity were visualized by immunohistochemistry for PLP and the human leukocyte antigen (HLA-DR) to visualize microglia/macrophages (see antibody details in Supplemental Table 2). Meningeal immune cells were identified by immunohistochemistry for CD3 to detect T cells, CD20 to detect B cells, and IBA1 and CD68 to detect myeloid cells (Supplemental Table 2).

Immunohistochemistry was performed as previously described (61, 62). Sections of 7 μm thickness were cut from FFPE tissue blocks, collected on Superfrost Plus glass slides (VWR international), and dried overnight at 37°C. Sections were deparaffinized in xylene (2 × 15 minutes) and rehydrated through a series (100%, 70%, 50%) of ethanol. Endogenous peroxidase activity was blocked by incubation in methanol (Merck KGaA) with 0.3% H_2_O_2_ (Merck KGaA) for 20 minutes at room temperature. Sections were then rinsed in PBS and pretreated with microwave antigen retrieval (3 minutes at 900 W followed by 10 minutes at 90 W) in either 0.05 M TBS, pH 7.6, or 10 mM Tris/1 mM EDTA buffer pH 9.0 (Supplemental Table 2).

Sections were incubated overnight at 4°C in the appropriate primary antibody (Supplemental Table 2) diluted in Normal Antibody Diluent (ImmunoLogic) and the next day with the BrightVision poly-HRP-anti Ms/Rb/Rt IgG biotin-free (diluted 1:1 in PBS, ImmunoLogic) for 30 minutes at room temperature. The immunostaining was visualized with DAB (Vector Laboratories) for 4 minutes at room temperature, and sections were counterstained with hematoxylin (Sigma Chemie GmbH), dehydrated in ethanol, and mounted with Pertex (Histolab).

### Classification of cortical GML type.

Cortical GMLs were classified based on the location of the demyelinating plaque, as previously described (1). Leukocortical (type I) lesions were contiguous with subcortical WML; intracortical (type II) lesions were confined to the cortex and often perivascular; and subpial (type III) lesions extended from the pial surface typically to cortical layers 2–4.

### Classification of subcortical WML type.

Subcortical WMLs lay underneath the cortical gray matter. Of note, periventricular lesions are not scored separately from other WMLs and are therefore included in this study as part of the assessment of subcortical WMLs. WMLs were classified as previously described (61, 63) into active, mixed active/inactive (also known as chronic active), inactive, or remyelinated. Active WMLs typically showed loss of LFB and PLP with HLA^+^ microglia/macrophages spanning the entire lesion area. Mixed active/inactive WMLs typically showed loss of LFB and PLP with a hypocellular and inactive lesion center, surrounded by a rim of activated HLA^+^ microglia/macrophages (Supplemental Figure 7). Inactive WMLs also showed loss of LFB and PLP, though their borders were sharply demarcated with few or no HLA^+^ microglia/macrophages (Supplemental Figure 8). Remyelinated lesions were identified by thinner myelin sheaths resulting in a paler PLP^+^ staining intensity.

### WML subtype calculations.

To calculate the proportions of WML subtypes, all available archived FFPE tissue blocks for each of the 27 patients with MS (range 5–72 blocks, median: 30 blocks per donor) were analyzed (see Supplemental Table 1). One section per block was used, and all lesions in the section that were visibly distinct were counted separately. The proportion of active lesions was defined as the number of active lesions/(the number of active + mixed active/inactive + inactive + remyelinated lesions). The proportion of mixed active/inactive lesions was defined as the number of mixed active/Inactive lesions/(the number of active + mixed active/inactive + inactive + remyelinated lesions). The proportion of inactive lesions was defined as the number of inactive lesions/(the number of active + mixed active/inactive + inactive + remyelinated lesions). The proportion of remyelinated lesions was defined as the number of remyelinated lesions/(the number of active + mixed active/inactive + inactive + remyelinated lesions).

### Quantification of meningeal inflammation.

Meningeal segments were randomly selected for imaging at 20× original magnification with a light microscope (Olympus BX41TF) connected to Cell D software (Olympus). Immune cells were quantified in meningeal areas that were adjacent to type III (subpial) GMLs and in areas adjacent to NAGM. A total of 71% ± 20% (mean ± SD) of intact meninges were available for scoring in the MS cohort, and 40% ± 8% (mean ± SD) of intact meninges were available for scoring in the nonneurological control cohort. CD20^+^ B cell counts were done over a total meningeal area of 47.53 mm^2^ from patients with MS, of which 37.02 mm^2^ was adjacent to GMLs and 10.51 mm^2^ was adjacent to NAGM; and 5.652 mm^2^ of meningeal area was adjacent to nonneurological control cortex. CD3^+^ T cell counts were done in a total meningeal area of 60.97 mm^2^ from patients, of which 48.3 mm^2^ was adjacent to GMLs and 12.67mm^2^ was adjacent to NAGM; and 6.807 mm^2^ of meningeal area was adjacent to nonneurological control cortex. IBA1^+^ cell count was done in a total meningeal area of 46.93 mm^2^ from patients, of which 35.16 mm^2^ was adjacent to GMLs and 11.77 mm^2^ was adjacent to NAGM; and 6.66 mm^2^ of meningeal area was adjacent to nonneurological control cortex. CD68^+^ cell count was done in a total meningeal area of 44.67 mm^2^ from patients, of which 30.07 mm^2^ was adjacent to GMLs and 14.6 mm^2^ was adjacent to NAGM; and 7.97 mm^2^ of meningeal area was adjacent to nonneurological control cortex. The meningeal area (in mm^2^) was measured using the “measurement” function of Image Pro Plus 7.0 imaging software (Media Cybernetics). Cell numbers were expressed as mean number per millimeter of intact meninges.

### Statistics.

All tests were performed using GraphPad Prism software 5.0. The variability of distribution was assessed by Shapiro-Wilk normality test. The nonnormally distributed data were analyzed by the nonparametric Mann-Whitney test (between 2 groups) or Kruskal-Wallis test followed by Dunn’s correction for multiple comparisons (between more than 2 groups). Results were considered significant when *P* was less than 0.05 at a 95% confidence interval.

### Study approval.

All postmortem human tissue was collected with written informed consent for the use of material and clinical data for research purposes, in compliance with ethical guidelines of the Vrije Universiteit and NBB, Amsterdam, Netherlands (reference 2009/148). In addition, the University of Toronto Research Ethics Board, Toronto, Canada, granted approval for conducting histology and correlation with clinical metadata on all postmortem human tissue (study 36850).

## Author contributions

SMA conducted the experiments and analyzed the data; NLF acquired data; HT, JLG, and IH advised on the project; IM and VR participated in the initial data acquisition; ABO and VR designed the study; and VR wrote the manuscript.

## Supplementary Material

Supplemental data

## Version 1 02/01/2022

In-Press Preview

## Version 2 03/08/2022

Electronic publication
