# Supplementary material for: Accumulation of meningeal lymphocytes correlates with white matter lesion activity in progressive multiple sclerosis
Source: JCI Insight. 2022 Mar 8;7(5):e151683. doi: 10.1172/jci.insight.151683 (PMC8983127; doi:10.1172/jci.insight.151683)
Supplement: Supplemental data [file jciinsight-7-151683-s129.pdf]

Accumulation of meningeal lymphocytes correlates with white matter lesion activity in progressive MS

Shanzeh M. Ahmed[1], Nina Fransen[2,3], Hanane Touil[4], Iliana Michailidou[5], Inge Huitinga[2,6], Jennifer L. Gommerman[1], Amit Bar-Or[4]*, Valeria Ramaglia[1]*

[1]Department of Immunology, University of Toronto, Toronto, Ontario M5S 1A8, Canada; [2]Department of Neuroimmunology, Netherlands Institute for Neuroscience, Meibergdreef 47, 1105 BA, Amsterdam, The Netherlands;

[3]Department of Pathology, University Medical Center Utrecht, Utrecht, The Netherlands

[4]Department of Neurology and Center for Neuroinflammation and Neurotherapeutics, Perelman School of Medicine, University of Pennsylvania, Philadelphia, PA, USA;

[5]Department of Clinical Genetics, Leiden University Medical Center, Einthovenweg 20, 2333ZC, Leiden, The Netherlands

[6]Swammerdam Institute for Life Sciences, University of Amsterdam, Amsterdam, The Netherlands

***Corresponding authors:**

Valeria Ramaglia, University of Toronto, Medical Sciences Building, Department of Immunology 1 King's College Circle, Toronto, ON, M5S 1A8, Canada. Tel: +1 416-978-4119

v.ramaglia@utoronto.ca

and

Amit Bar-Or, Department of Neurology, Perelman School of Medicine, University of Pennsylvania, PCAM building, Room 763, 3400 Civic Center Blvd, Philadelphia, PA, USA, 19104. Tel: +1 267-591-0136. amitbar@pennmedicine.upenn.edu

Supplemental Figures:

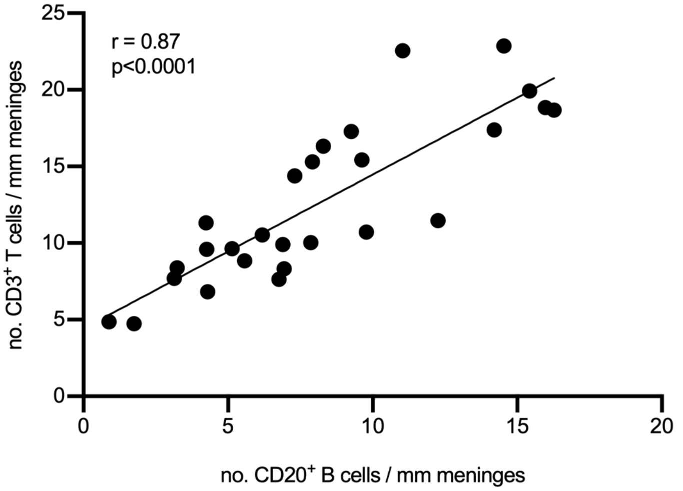

Supplementary figure 1. The density of meningeal T cells positively correlates with the density of meningeal B cells in MS. Spearman correlation coefficient between meningeal $CD3^+$ T cell count and meningeal $CD20^+$ B cell count of MS donors. Each data point represents the mean meningeal cell count in all fields analyzed per case.

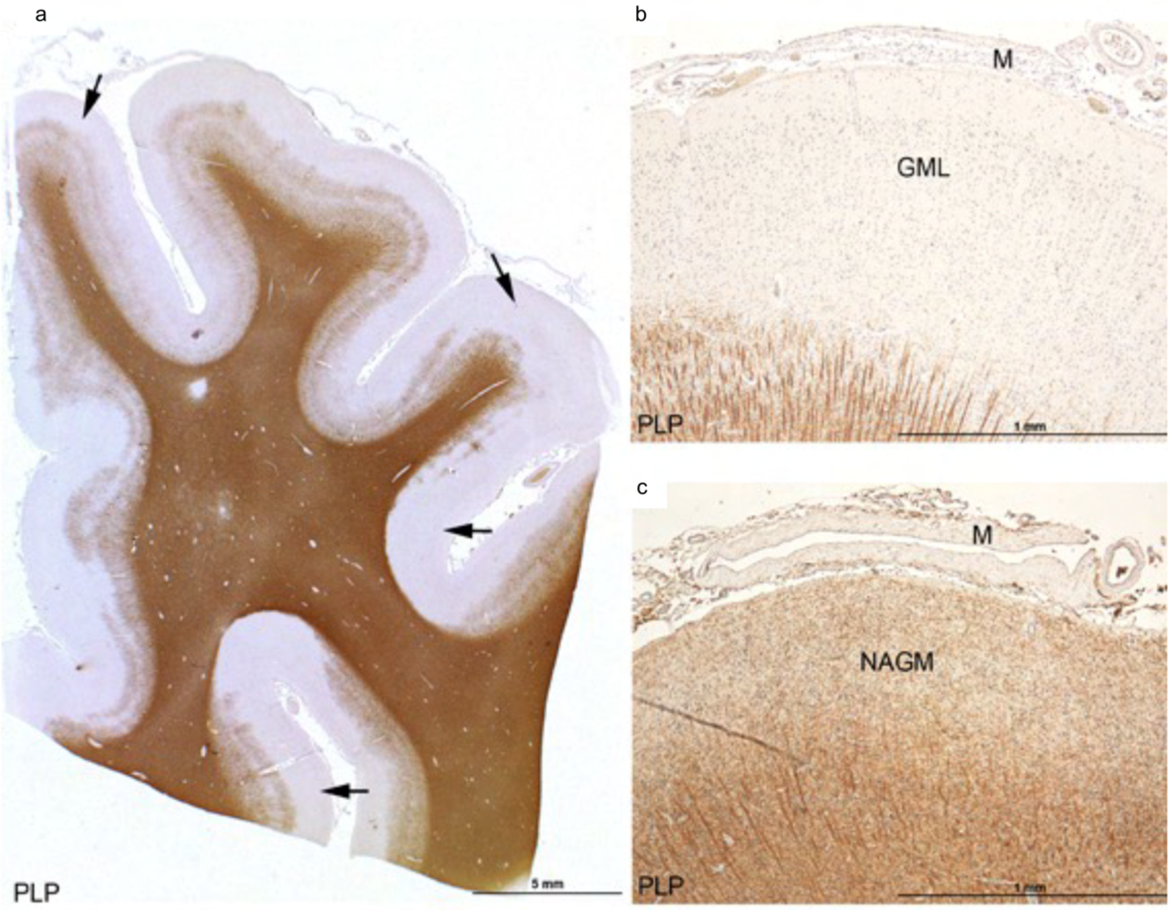

Supplementary figure 2. Cortical subpial demyelination in MS. a-c. Immunohistochemical staining with anti-myelin proteolipid protein (PLP) of MS cortex with subpial grey matter demyelination (arrows in **a**). Close up of MS cortex showing a subpial (type III) grey matter lesion (GML) (**b**) or normal appearing grey matter (NAGM) (**c**) adjacent to meninges (M). In **a**, scale bar represents 5mm; In **b**, **c**, scale bars represent 1mm.

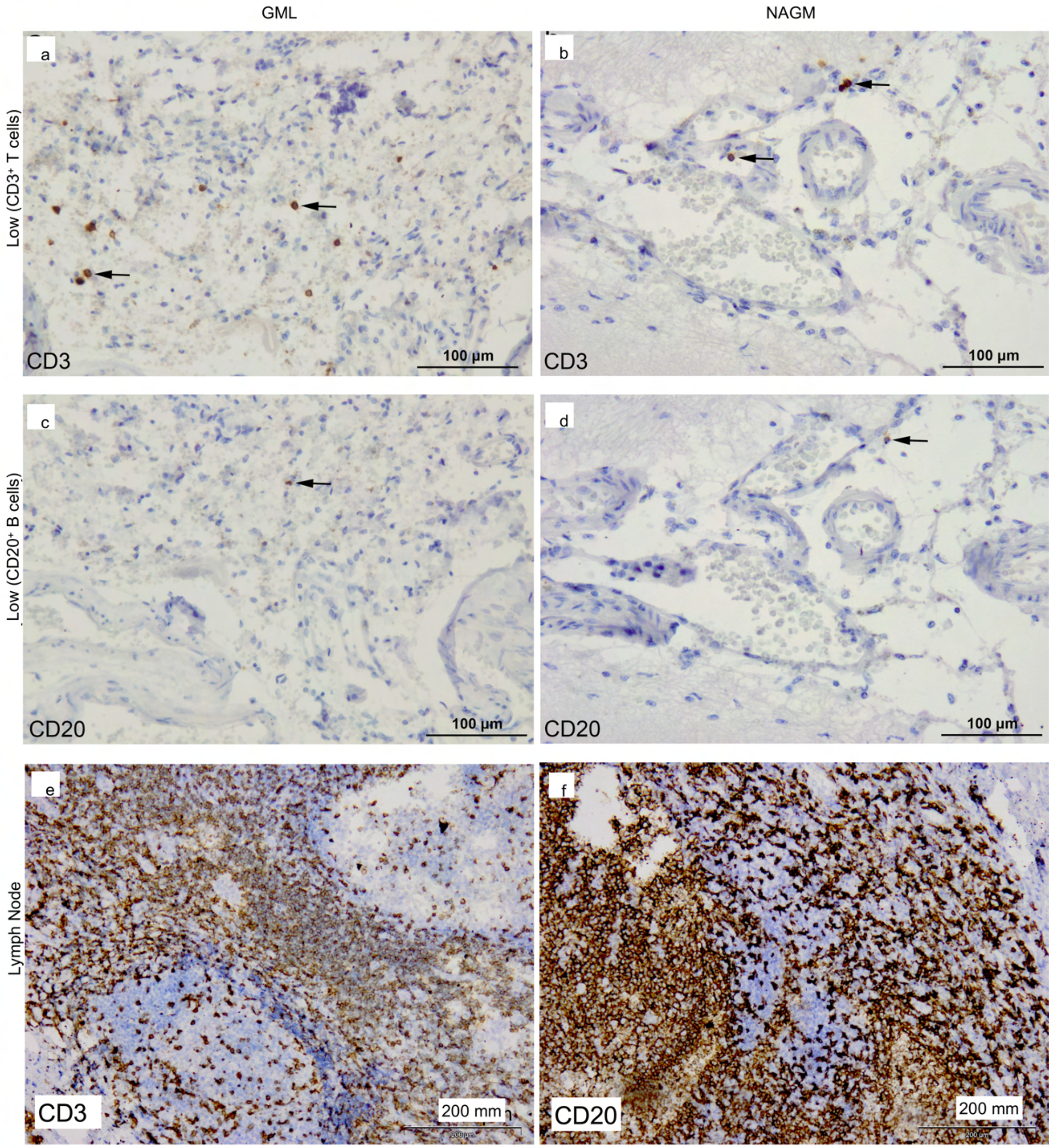

Supplementary figure 3. Meningeal T cells and B cells in the 'low' donor group are not enriched in proximity of cortical subpial demyelinated lesions. Representative immunohistochemical staining for CD3 (**a and b, arrows**) and CD20 (**c and d, arrows**) in meninges adjacent to a subpial grey matter lesion (GML) or adjacent to normal appearing gray matter (NAGM) in MS donors with low $CD3^+$ or $CD20^+$ meningeal cell count. **e., f.** Representative immunostaining for CD3 and CD20 in Lymph Nodes used as technical positive controls. In **a-d**, scare bars represent 100μm. In **e, f**, scare bars represent 200μm.

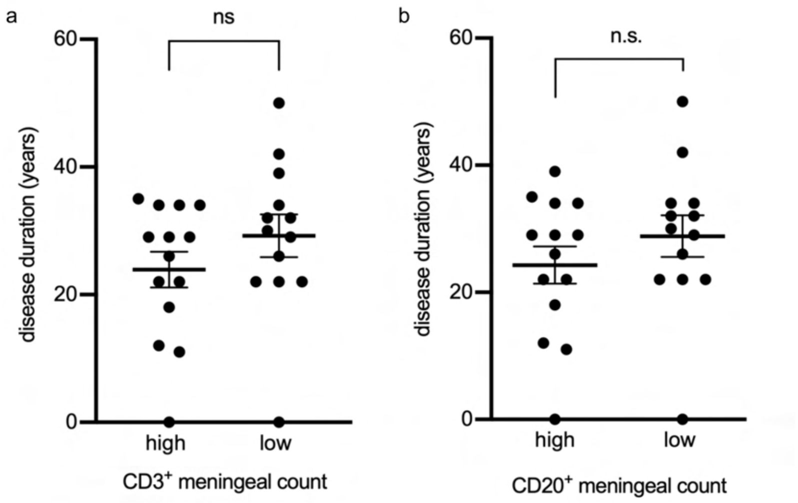

Supplementary figure 4. Enrichment of meningeal T cells and B cells is not linked to disease duration. Disease duration in MS donors with high vs low (**a**) $CD3^+$ or (**b**) $CD20^+$ meningeal cell count. Data shown as mean±SD. Statistically significant differences were tested by the non-parametric Mann Whitney test ($p<0.05$).

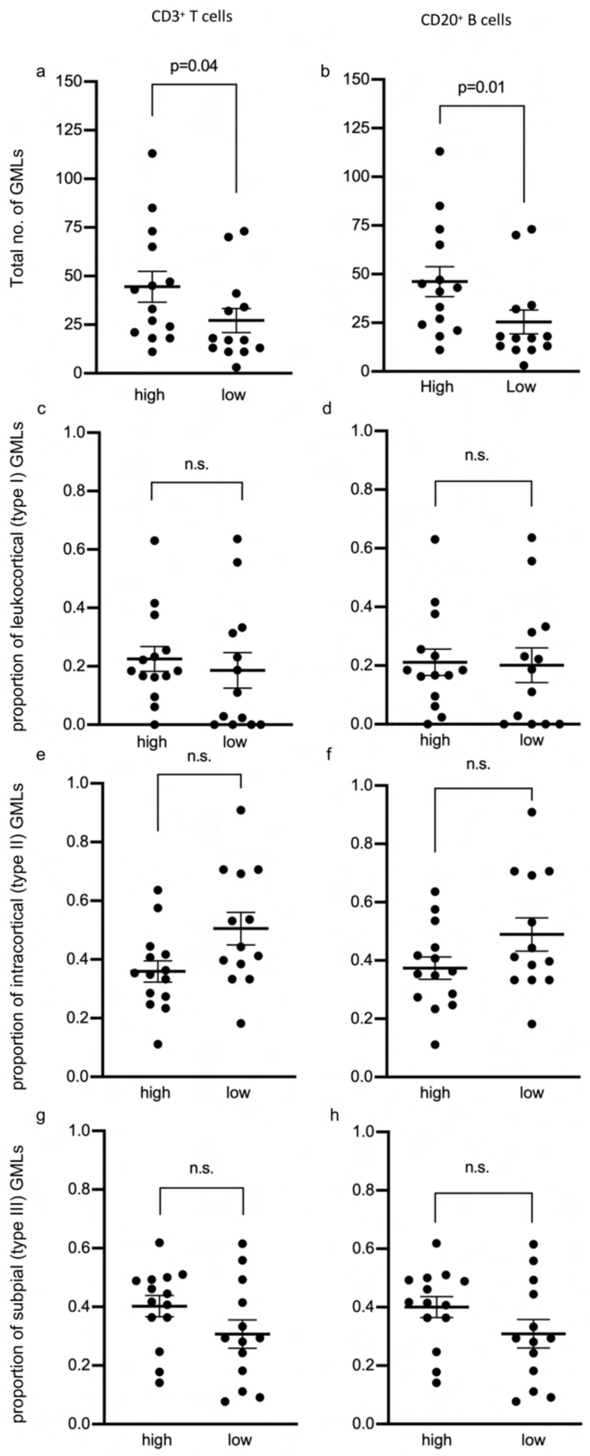

Supplementary figure 5. Enrichment of meningeal T cells and B cells is not linked to the proportion of cortical subpial demyelinated grey matter lesions. Quantification of (**a, b**) number of cortical grey matter lesions (GMLs), (**c, d**) proportion of leukocortical (type I) GMLs, (**e, f**) proportion of intracortical (type II) GMLs and (**g, h**) proportion of subpial (type III) GMLs in MS donors with high vs low meningeal $CD3^+$ T cells (**a, c, e, g**) or $CD20^+$ B cell (**b, d, f, h**) count. Each data point represents the proportion of GMLs in all tissue blocks analyzed per case. Data shown as mean±SD. Statistically significant differences were determined by the non-parametric Mann Whitney test.

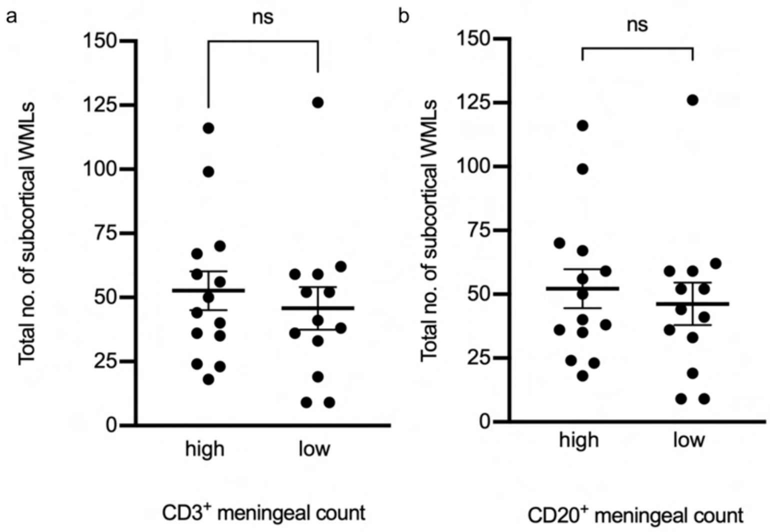

Supplementary figure 6. Enrichment of meningeal T cells and B cells is not linked to the total number of subcortical white matter lesions. Total number of subcortical white matter lesions in MS donors with high vs low (**a**) $CD3^+$ or (**b**) $CD20^+$ meningeal cell count. Data shown as mean±SD. Statistically significant differences were tested by the non-parametric Mann Whitney test ($p<0.05$).

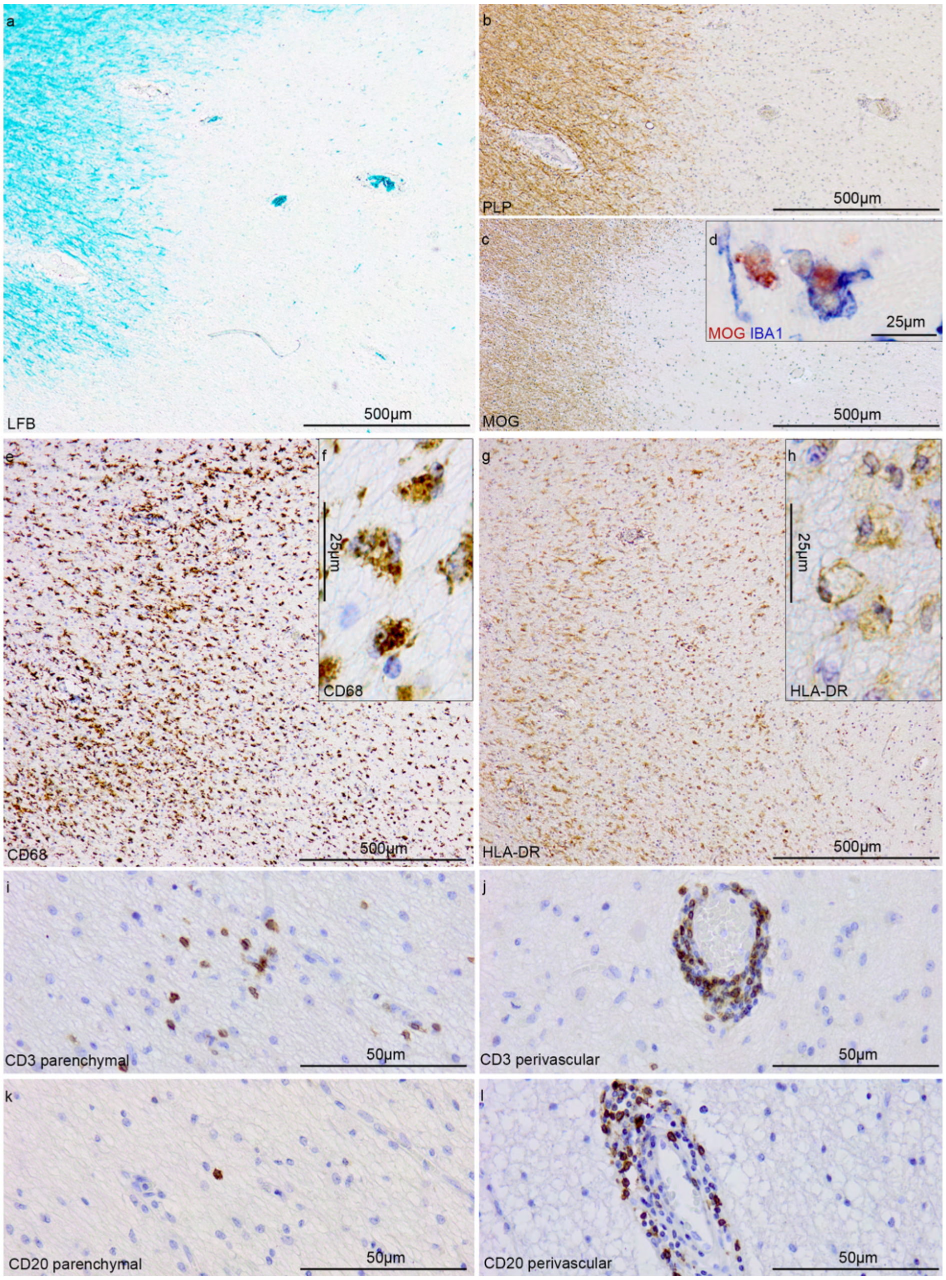

Supplementary figure 7. Characterization of mixed active-inactive white matter lesions (WLM) in progressive MS cases. A mixed active-inactive WML in progressive MS case, showing loss of luxol fast blue (LFB) staining (**a**), loss of immunoreactivity for the proteolipid protein (PLP, in **b**) and loss of immunoreactivity for the myelin oligodendrocyte glycoprotein (MOG, in **c**) within the lesions, with intracellular inclusions of MOG+ myelin products inside ionized calcium-binding adapter molecule-1 (IBA1)+ macrophages at the lesion edge (**d**), indicative of demyelinating activity. Immunoreactivity for the CD68 lysosomal marker is abundant throughout the lesion and accumulates at the lesion edge (**e**) in a staining pattern consistent with enlarged lysosomes within phagocytic cells (**f**). Human leukocyte antigen (HLA-DR)+ myeloid cells accumulate at the lesion edge (**g**) and show enlarged morphology (**h**), consistent with the active phenotype. CD3+ T cells are found in the parenchyma (**i**) and as perivascular "cuffs", expanding within the Virchow-Robbin space of capillaries (**j**), located within the lesion or at the peri-lesional areas. CD20+ B cells are occasionally found in the parenchyma (**k**) and more often observed as perivascular "cuffs" (**l**), located within the lesion or at the peri-lesional areas.

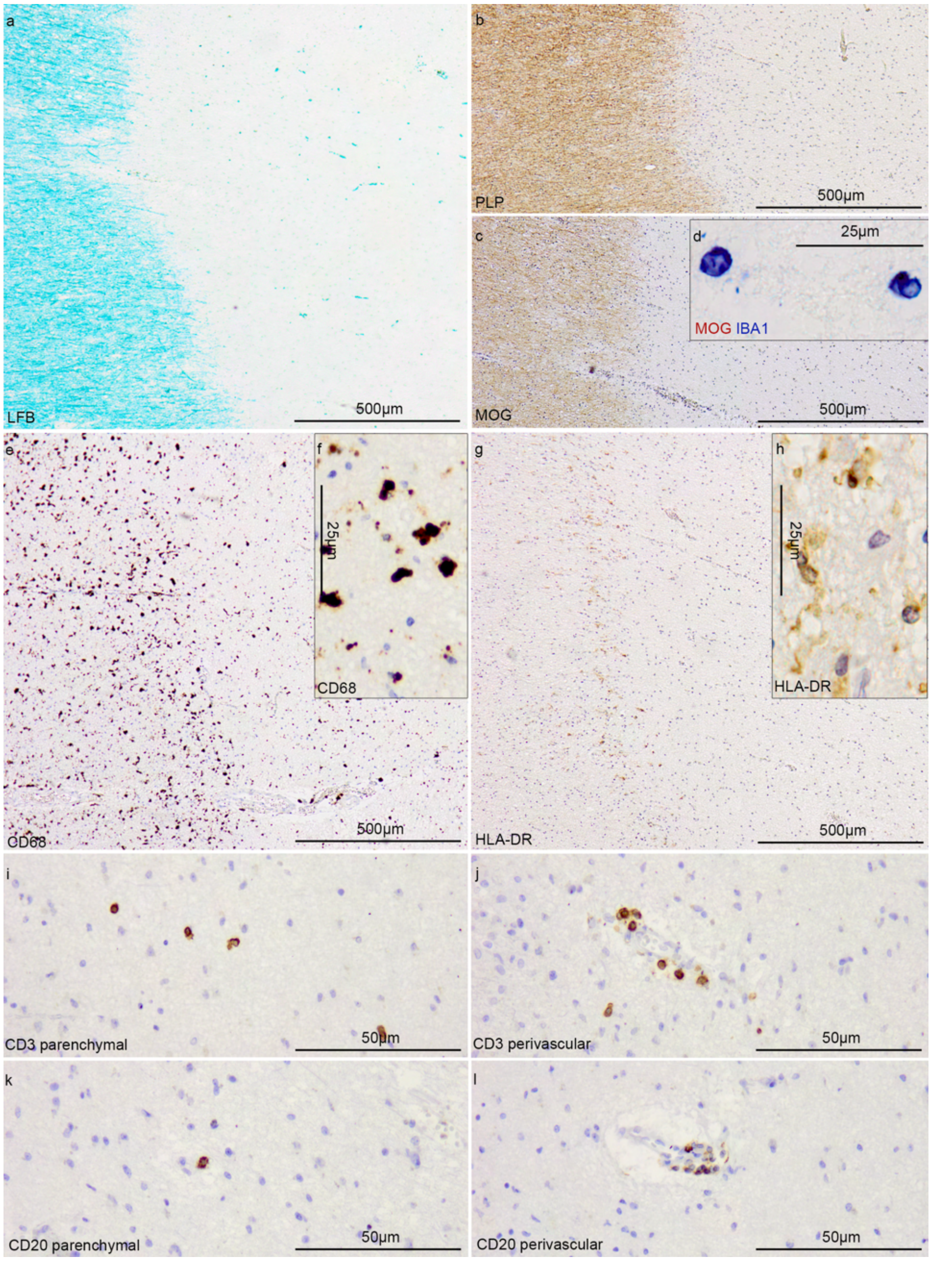

Supplementary figure 8. Characterization of inactive WML in progressive MS cases. Inactive WML in a progressive MS case, showing loss of luxol fast blue (LFB) staining (**a**), loss of immunoreactivity for the proteolipid protein (PLP, in **b**) and loss of immunoreactivity for the myelin oligodendrocyte glycoprotein (MOG, in **c**) with no evidence of intracellular inclusions of MOG+ myelin products inside ionized calcium-binding adapter molecule-1 (IBA1)+ macrophages (**d**), indicative of no demyelinating activity. Immunoreactivity for the CD68 lysosomal marker is scarce within the lesion and more obvious at the lesion edge (**e**) in a staining pattern consistent with inactive, gliotic cells (**f**). A paucity of human leukocyte antigen (HLA-DR)+ microglia can be found at the lesion edge (**g**) and show small ramified morphology (**h**), consistent with the inactive, resting phenotype. (**i-l**) CD3+ T cells and CD20+ B cells are occasionally found in the parenchyma (**i, k**) and perivascular space (**j, l**), located within the lesion or at the peri-lesional area.

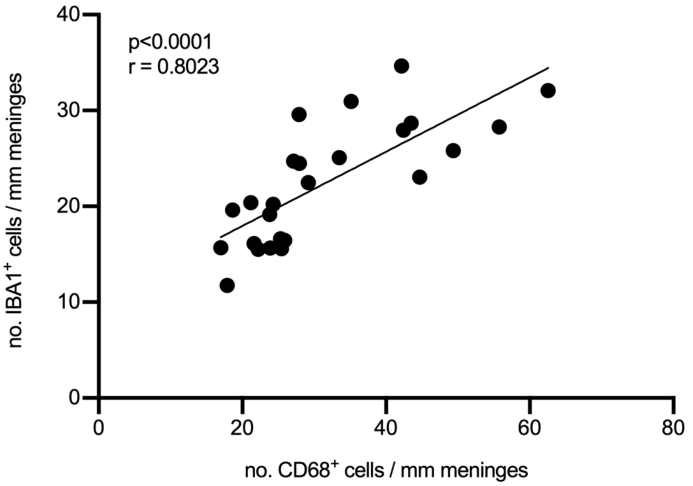

Supplementary figure 9. The density of meningeal IBA1$^+$ myeloid cells positively correlates with the density of meningeal CD68$^+$ cells in MS. Spearman correlation coefficient between meningeal IBA1$^+$ myeloid cell count and meningeal CD68$^+$ cell count of MS donors. Each data point represents the mean meningeal cell count in all fields analyzed per case.

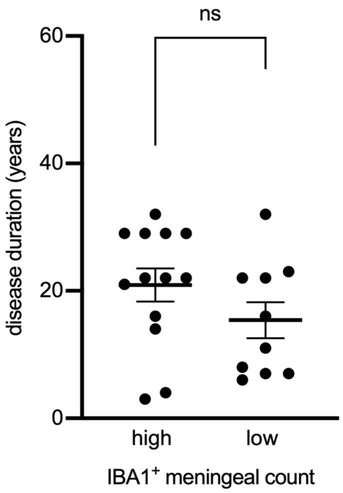

Supplementary figure 10. Enrichment of meningeal myeloid cells is not linked to disease duration. Disease duration in MS donors with high vs low IBA1$^+$ meningeal cell count. Data shown as mean±SD. Statistically significant differences were determined by the non-parametric Mann Whitney test ($p<0.05$).

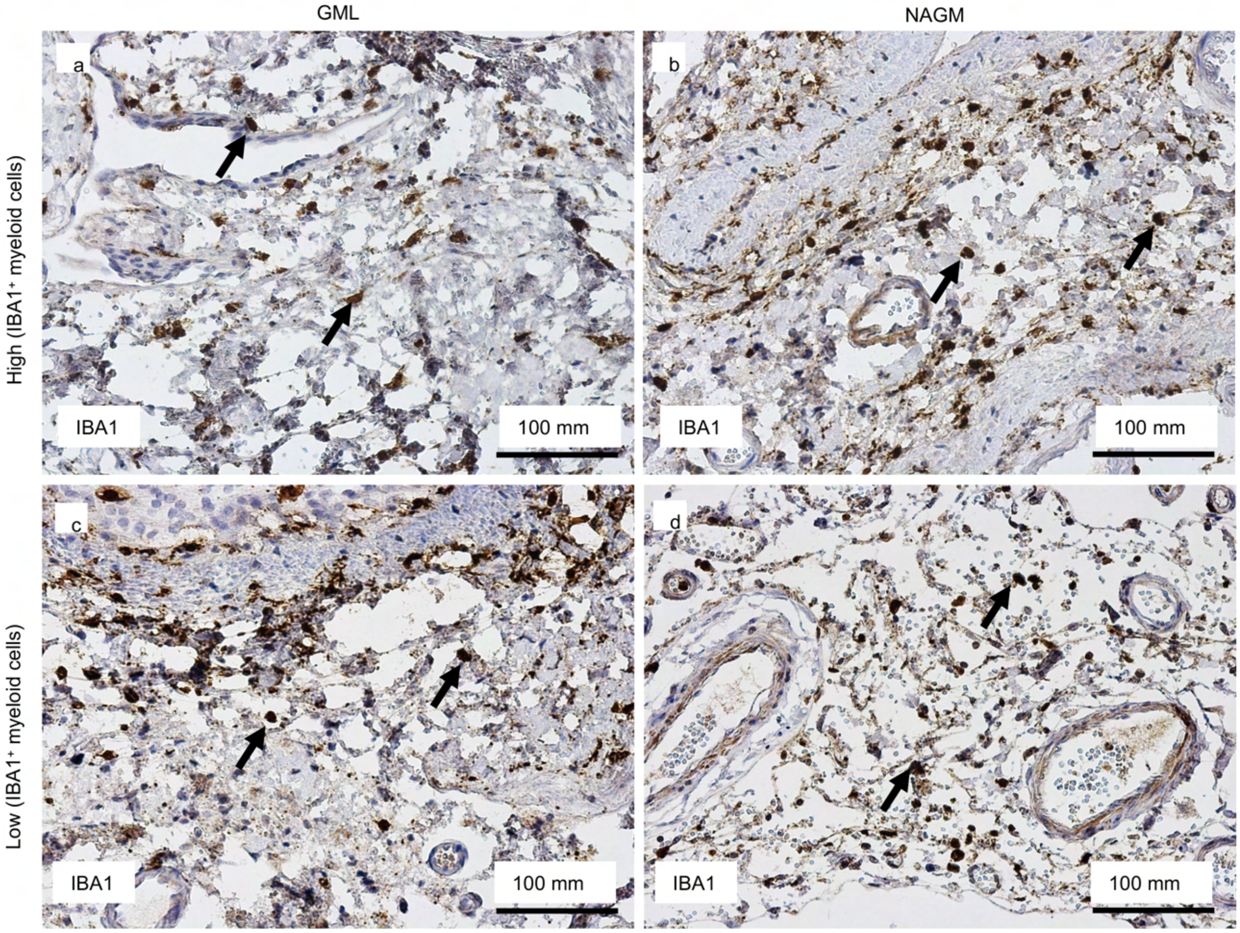

Supplementary figure 11. Meningeal myeloid cells are not topographically linked to subpial demyelination in MS. Representative immunohistochemical staining for IBA1 (arrows) in meninges adjacent to a subpial grey matter lesion (GML) or adjacent to normal appearing gray matter (NAGM) in MS donors with high (**a, b**) or low (**c, d**) meningeal $IBA1^{+}$ myeloid cell count. In **a-d**, scare bars represent 100μm.

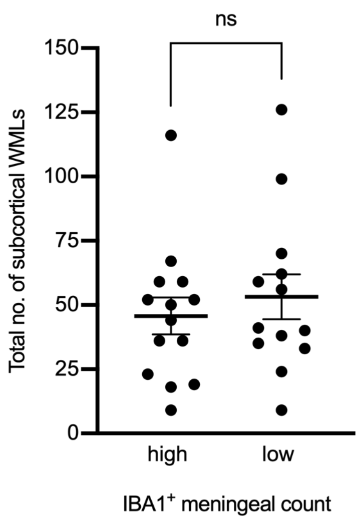

Supplementary figure 12. Enrichment of meningeal myeloid cells is not linked to the total number of subcortical white matter lesions. Total number of subcortical white matter lesions in MS donors with high vs low IBA1^{+} meningeal cell count. Data shown as mean±SD. Statistically significant differences were determined by the non-parametric Mann Whitney test ($p<0.05$).

Supplemental Tables:

Supplementary table 1. Donor demographics

Case	Sex	Age (years)	PMD (h:min)	Type of MS	DD (years)	No. tissue blocks analysed for lesion characterization	COD
MS							
2010-034	F	57	08:40	SPMS	26	41	Respiratory insufficiency to (uro)sepsis
2015-006	F	57	10:40	SPMS	29	30	Euthanasia
2015-011	F	57	07:30	SPMS	34	27	Euthanasia
2013-047	F	48	11:50	PPMS	22	32	Respiratory failure with end stage MS
2012-084	M	63	08:15	SPMS	30	23	Pneumonia, cachexia and dehydration
2015-047	F	50	09:05	SPMS	18	23	Euthanasia
2015-082	F	47	08:35	PPMS	29	34	Aspiration pneumonia
1996-039	F	57	05:45	PPMS	22	15	Sepsis
2004-017	F	41	08:25	SPMS	11	8	Natural death
2010-025	M	51	11:00	SPMS	>12	38	Exact cause unknown, infection 2 days prior to death
2010-040	F	57	08:40	PPMS	29	31	Euthanasia
2011-080	F	56	08:25	SPMS	34	27	Respiratory insufficiency secondary to pneumonia
2011-089	M	64	07:30	-	-	28	-
1997-006	F	62	06:45	PPMS	29	5	Cardiac asthma
2003-020	F	68	07:30	SPMS	39	23	Bronchitis/ aspiration pneumonia
2012-113	M	59	10:45	RRMS	22	34	Euthanasia
2002-023	F	75	08:00	SPMS	42	32	Pneumonia
2008-021	M	55	06:20	PPMS	32	33	Respiratory insufficiency
2015-070	F	77	10:05	SPMS	26	18	Cardiovascular event and dehydration
2014-058	F	74	07:50	SPMS	50	72	Euthanasia
2012-113	M	59	10:45	RRMS	22	34	Euthanasia
2011-100	F	71	07:05	SPMS	34	26	Cachexia with slowly progressive MS and metastatic breast cancer
2012-045	F	81	09:35	PPMS	22	25	Cardiac asthma
2012-057	F	81	04:35	SPMS	34	30	Aspiration pneumonia
2012-078	M	58	09:15	-	-	30	-
2012-122	M	60	07:30	PPMS	32	30	Respiratory failure due to pneumonia
2014-007	F	51	09:45	SPMS	35	34	Euthanasia
Non-neurological Controls							
2011-069	M	49	06:15	-	-	-	Euthanasia with Hopkin's lymphoma
2009-021	F	99	04:15	-	-	-	-
1994-114	M	63	26:45	-	-	-	Pulmonary infection
2010-070	F	60	07:30	-	-	-	Infection e.c.i
2005-019	M	74	05:00	-	-	-	-
1998-061	F	64	06:00	-	-	-	Cachexia
2005-034	M	56	14:00	-	-	-	Heart failure
1994-119	F	51	7:40	-	-	-	-
2003-002	M	51	7:44	-	-	-	-

MS,multiple sclerosis; F, Female; M, Male; PMD, post-mortem delay; DD, disease duration; h:min, hours:minutes; COD, cause of death.

Supplementary table 2. Primary antibodies, dilution, source

Antigen	Clone	Dilution/Concentration	Source
Proteolipid protein (PLP)	Monoclonal (plpc1)	1:3000[a]	Serotec (Puchheim, Germany)
CD3	Monoclonal (SP7)	1:500[b]	Dako (Glostrup, Denmark)
CD20	Monoclonal (L26)	1:1000[b]	Dako
IBA1	Monoclonal (EPR16589)	1:8000[c]	Abcam
CD68	Monoclonal (PGM1)	1:200[b]	Dako
Human leukocyte antigen (HLA)-DR	Monoclonal (CR3/43)	8.6µg/ml[a]	Dako

Antigen retrieval of paraffin sections was performed by heat in [a] 0.05 M Tris buffered saline pH 7.6; [b] 10 mM Tris/1 mM EDTA buffer pH 9 or 10mM Sodium Citrate pH 6.0[c]
